# Supplementary figures and images for: Identification of significant genes signatures and prognostic biomarkers in cervical squamous carcinoma via bioinformatic data
Source: PeerJ. 2020 Dec 2;8:e10386. doi: 10.7717/peerj.10386 (PMC7718800; doi:10.7717/peerj.10386)

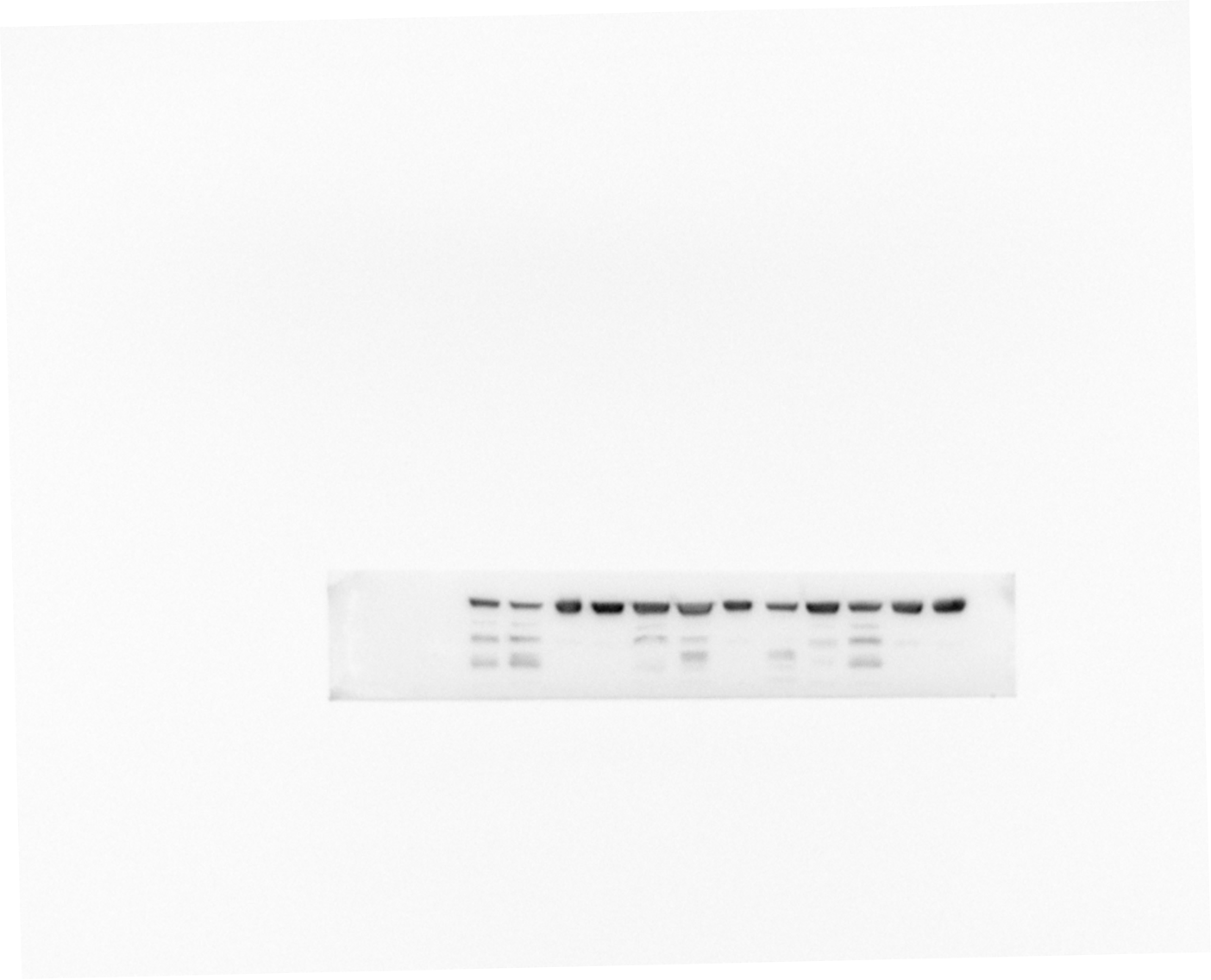

Supplement: Supplemental Information 2 [file peerj-08-10386-s002.zip › ACTIN.png]

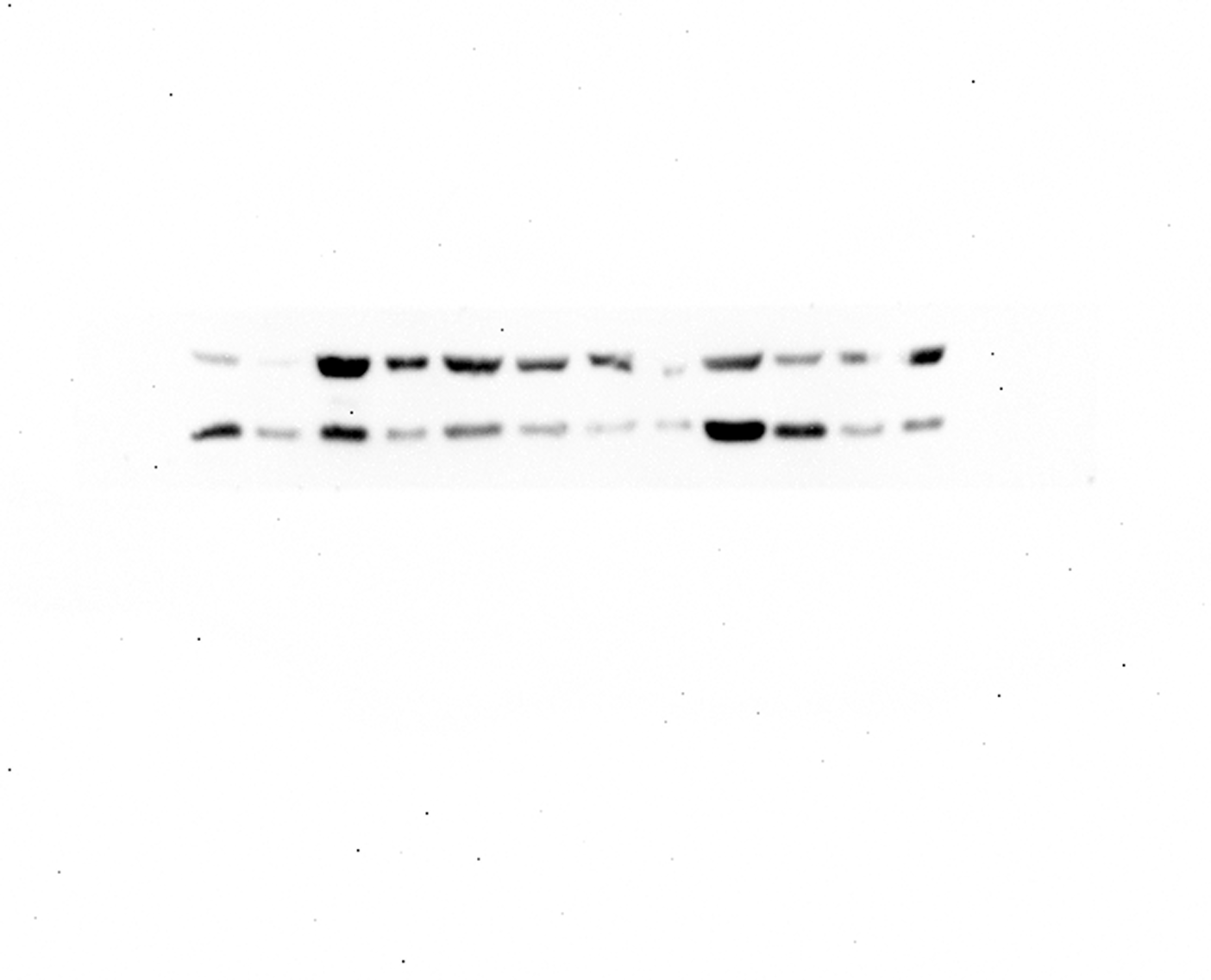

Supplement: Supplemental Information 2 [file peerj-08-10386-s002.zip › RFC4.png]
